# An evolutionary method for learning HMM structure: prediction of protein secondary structure

**DOI:** 10.1186/1471-2105-8-357

**Published:** 2007-09-21

**Authors:** Kyoung-Jae Won, Thomas Hamelryck, Adam Prügel-Bennett, Anders Krogh

**Affiliations:** 1 Bioinformatics Centre, Department of Molecular Biology, University of Copenhagen, Ole Maaloes Vej 5, DK-2200 Copenhagen, Denmark; 2 School of Electronics and Computer Science, University of Southampton, SO17 1BJ, UK; 3 Department of Chemistry & Biochemistry, UCSD, 9500 Gilman Drive, Mail Code 0359, La Jolla, CA, 92093-0359, USA

## Abstract

**Background:**

The prediction of the secondary structure of proteins is one of the most studied problems in bioinformatics. Despite their success in many problems of biological sequence analysis, Hidden Markov Models (HMMs) have not been used much for this problem, as the complexity of the task makes manual design of HMMs difficult. Therefore, we have developed a method for evolving the structure of HMMs automatically, using Genetic Algorithms (GAs).

**Results:**

In the GA procedure, populations of HMMs are assembled from biologically meaningful building blocks. Mutation and crossover operators were designed to explore the space of such Block-HMMs. After each step of the GA, the standard HMM estimation algorithm (the Baum-Welch algorithm) was used to update model parameters. The final HMM captures several features of protein sequence and structure, with its own HMM grammar. In contrast to neural network based predictors, the evolved HMM also calculates the probabilities associated with the predictions. We carefully examined the performance of the HMM based predictor, both under the multiple- and single-sequence condition.

**Conclusion:**

We have shown that the proposed evolutionary method can automatically design the topology of HMMs. The method reads the grammar of protein sequences and converts it into the grammar of an HMM. It improved previously suggested evolutionary methods and increased the prediction quality. Especially, it shows good performance under the single-sequence condition and provides probabilistic information on the prediction result. The protein secondary structure predictor using HMMs (P.S.HMM) is on-line available http://www.binf.ku.dk/~won/pshmm.htm. It runs under the single-sequence condition.

## Background

Prediction of protein secondary structure is an important step towards understanding protein structure and function from protein sequences. This task has attracted considerable attention and consequently represents one of the most studied problems in bioinformatics. Early prediction methods were developed based on stereochemical principles [1] and statistics [2,3]. Since then the prediction rate has steadily risen due to both algorithmic development and the proliferation of the available data. The first machine learning predictions of secondary structure were done using neural networks [4,5]. Later methods using neural networks include PHD [6], PSIPRED [7], SSpro [8], SSpro8 [9] and YASPIN [10]. Support vector machines have also been used and show promising results [11]. Recently, the prediction accuracy has been improved by cascading a second layer of support vector machines [12,13]. The currently used machine learning methods typically improve their performance by combining several predictors and using evolutionary information obtained from PSI-BLAST [14]. Combining results from different predictors has been shown to improve the performance of secondary structure prediction [15,16].

Even though Hidden Markov Models (HMMs) have been successfully applied to many problems in biological sequence modelling, they have not been used much for protein secondary structure prediction. Asai et al. suggested the first HMM for the prediction of protein secondary structure [17]. Later, an HMM with a hierarchical structure was suggested [18]. However, both predictors had limited accuracy.

HMMSTR [19] is a successful HMM predictor for this problem. It was constructed by identifying recurring protein backbone motifs (called invariant/initiation sites or I-sites) and representing them as a Markov chain. Consequently, the topology of HMMSTR can be interpreted as a description of the protein backbone in terms of consecutive I-sites. YASPIN [10], which is one of the most recent methods, builds on a combination of hidden Markov models and neural networks [20].

In this paper, we report a new method for optimizing the structure of HMMs for secondary structure prediction. Over the last couple of years we have developed a method for optimizing the structure of HMMs automatically using Genetic Algorithms (GAs) [21,22]. In previous work, we applied this method to promoter finding in DNA. Here, we use the evolutionary method to optimize the structure of an HMM for secondary structure prediction. During the evolutionary optimization, the HMM's structure is assembled from biologically meaningful building blocks [22]. Hence, we call our evolutionary method Block-HMM. The evolved HMM using the Block-HMM remodels the training protein sequences and shows the prediction probability of the secondary conformations calculated for each amino acid.

In the literature, we have found a few HMM structure learning methods. Stolcke developed a state merging method, which starts from an HMM with a large number of states [23]. On the other hand, a state splitting method was suggested in [24,25]. A structure evolving method using GAs was first suggested to change the structure of a TATA box HMM [26]. Later, they upgraded the HMM structure learning method considering statistical significance [27]. A structure evolving method using a genetic programming was also suggested, in which the HMM structures is represented by probabilistic trees [28,29]. The evolving method was also applied to protein secondary structure prediction. Thomsen suggested a GA very similar to Yada et al. and achieved 49% prediction accuracy [30].

Our structure learning method is different from previous methods in that we use block models inspired by HMM applications used in biological sequence analysis. Instead of crossing over arbitrary number of states, we cross a number of blocks over. This enables different number of states to be exchanged through the crossover operation. Mutation occurs in a limited area that adding or deleting transitions do not break the property of blocks. As a result, our approach makes use of characteristics of HMM modularity more strategically than previously suggested genetic methods. Genetic programming methods [28,29] encode HMM networks with probabilistic trees. Linguistic representations were derived from each particular HMM topology. Similar to genetic programming method, our approach encodes several types of blocks into linguistic forms [22]. The basic shapes of linguistic blocks are different each other in both of the methods. The encoding differences effect the searching space of a topology evolution. It also suggests that various types of topological encoding may be useful for other problems.

We analyze one of the evolved HMM structure under the single-sequence condition. We also test it under the multiple-sequence condition after designing a whole predictor using an ensemble of three independently trained predictors as well as simple neural networks.

## Results

### Block-HMM for labelled sequences

**Figure 1:**
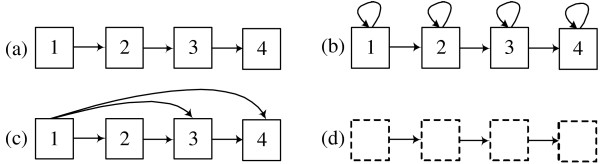
**HMM blocks that compose the whole HMM structure**. (a) linear block (b) self-loop block (tying is optional) (c) forward-jump block (tying is optional) (d) zero block.

Block-HMM restricts its search to a subset of HMM topologies made up of blocks of states. Each block is assigned a label that corresponds to one of the three secondary structure classes. The states that make up the blocks emit amino acid symbols. Secondary structure prediction is done by inferring the values of the hidden states for a given amino acid sequence, and examining the secondary structure labels of the blocks these states belong to. Four types of blocks are used: linear, self-loop, forward-jump blocks and zero blocks (figure 1).

Linear blocks consist of *N *states (labelled from 1 to *N*) where state *n *is only connected to state *n *+ 1 (with 1 ≤ *n *<*N*). Self-loop blocks are linear blocks in which each state has an additional loop to itself. A forward-jump block is a linear block where the first state is also connected to the last *M *states (with 1 <= *M *<*N*). Zero blocks are empty blocks with no states: they can replace other block types during the GA procedure and thus allow the exploration of simpler topologies.

The self-loop and forward-jump blocks can be either tied (in the figures, tied blocks are shaded) or untied. When a block is tied all the emission and transition probabilities of states inside the block are equal. In the case of linear blocks we did not consider tying because tying a linear blocks is equivalent to a single-state self-loop block.

**Figure 2:**
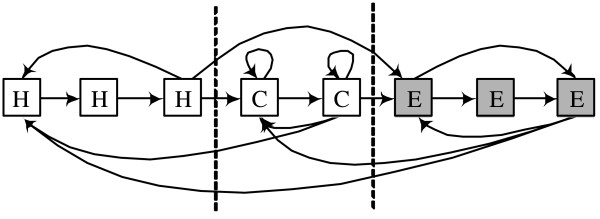
**An example of an HMM composed of blocks resulting from the Block-HMM procedure**. Three blocks are used in this model and all the blocks are fully connected to each other. The blocks are divided by dotted lines. The states in tied blocks are shaded in grey.

The various blocks can model different types of sequence fragments. A linear block can model a particular conserved sequence pattern. The self-loop block can model a sequence of any length, while the forward-jump block can be used to represent subsequences with varying length up to some fixed length. Initially, the blocks are fully linked to form HMM architectures. In this context, fully linked means that the end state of each block is connected to the starting states of all other blocks and itself. Each block is labelled with one of the three protein structure classes 'H' (helix), 'E' (strand), or 'C' (coil). Figure 2 shows a simple example of HMM structure. The HMM structure is composed of 3 blocks. From the left it has blocks labelled with 'H','C' and 'E'. Each block also can be tied. After training, most of the transition probabilities are close to zero, resulting in a final structure that is typically much simpler than the fully connected HMM shown in the figure.

### Genetic operators for Block-HMM

Genetic algorithms evolve a population of solutions with genetic operators. Inside the genetic cycle, genetic operators select members of the population (called parents) and evolve them to produce new members (called children). New children after the genetic operators along with the remaining old members in a population are evaluated to calculate fitness. According to the fitness selection procedure select a number of members in a population for the next genetic cycle.

**Figure 3:**
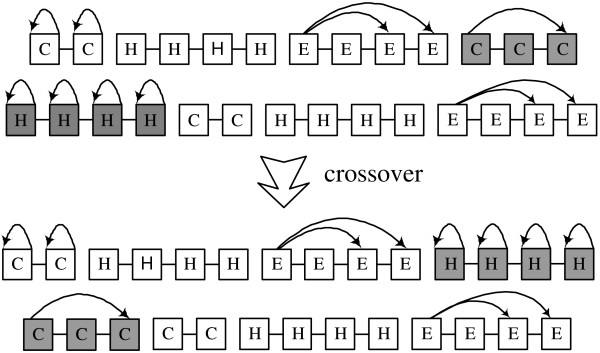
**Crossover in Block-HMM**. Crossover swaps the HMM states without changing the properties of an individual HMM block. Here, the last block of the first child crosses with the first block of the second child. To simplify the diagram, transitions between blocks are not shown.

**Figure 4:**
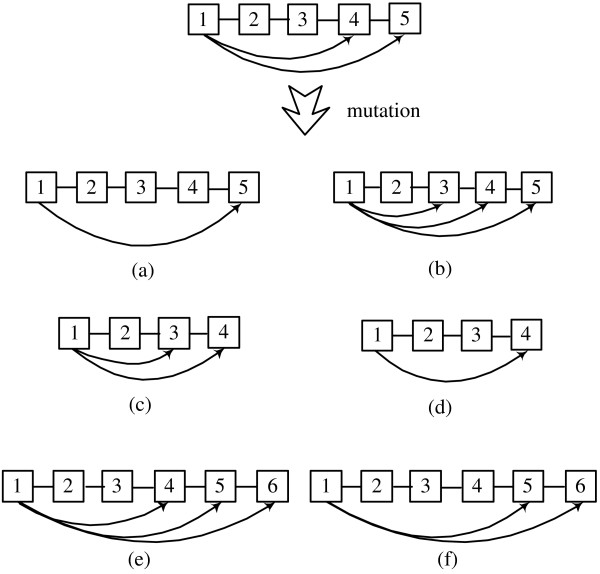
**Mutation in Block-HMM**. Six possible types of mutations from a 5-state forward-jump block: (a) a transition from the first to the fourth state is deleted (b) a transition from the first to the third state is added (c) the second or the third state is deleted (d) the fourth state is deleted (e) a state is added between the fourth and the fifth state (f) a state is added between the first and the fourth state.

We used three genetic operators in Block-HMM: crossover, mutation and type-mutation. The number of blocks is kept fixed but the number of the states of an HMM can be changed by the genetic operators. Crossover swaps a number of blocks in two parents to create two children. The crossover points and the number of blocks are chosen randomly. Figure 3 shows an example of the crossover scheme. The last block of the first child crosses with the first block of the second child. To simplify the diagram, transitions between blocks are not shown here. The crossover operator enables HMMs to exchange states without breaking basic blocks. Several blocks can be chosen to be crossed, which allows GA to search broad area of solution space. Mutations can take place inside any block of the HMM. A forward-jump block can have 6 different types of mutation, which are illustrated in figure 4. It can delete or insert transition (figure 4(a),(b)), delete one state (figure 4(c),(d)), and add one state (figure 4(e),(f)). For linear and self-loop blocks, only adding and deleting a state are possible.

**Figure 5:**
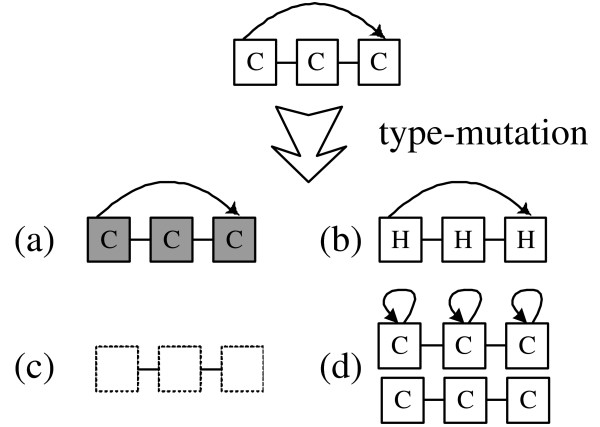
**Type-mutation in Block-HMM**. A forward jump block is type mutated (a) to a tied block (b) to a block with a different label (c) to a zero block (d) to a self loop block or a linear block.

In addition to changing the length of a block and its transitions, we also allow another form of mutation, called *type-mutation*, that changes the type or label of a block. Type-mutation to a zero block is also allowed (figure 5). When a type mutation transforms the type of a block, new transition probabilities are generated randomly. Self-loop and forward-jump blocks can type-mutate between tied and untied versions. Zero-blocks can be type-mutated to any of the other block forms.

We ran the GA that hybridize the parameter learning method with these genetic operators that train the structure of HMMs. The detailed description of the whole procedure is on Methods.

### Analysis of the evolved HMM

#### The evolved model

**Figure 6:**
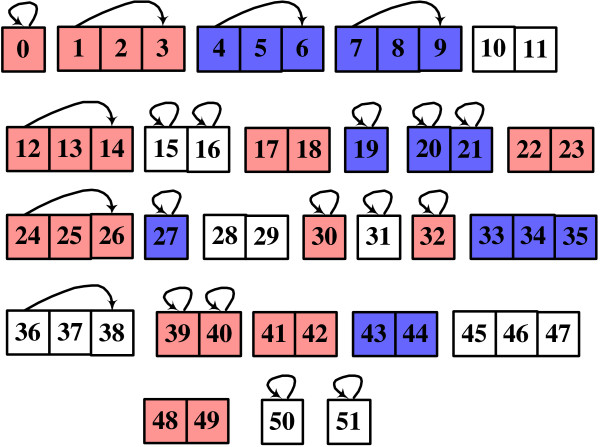
**The best HMM topology**. The best HMM topology evolved using Block-HMM. It is composed of 26 non-zero blocks and 52 states. Transitions between blocks are not shown here (including the transition from a block to itself). On each state a label is assigned ('H' for helices, 'E' for *β*-strands and 'x' for coils). Helix states are red colored and *β*-strand states are blue colored.

**Figure 7:**
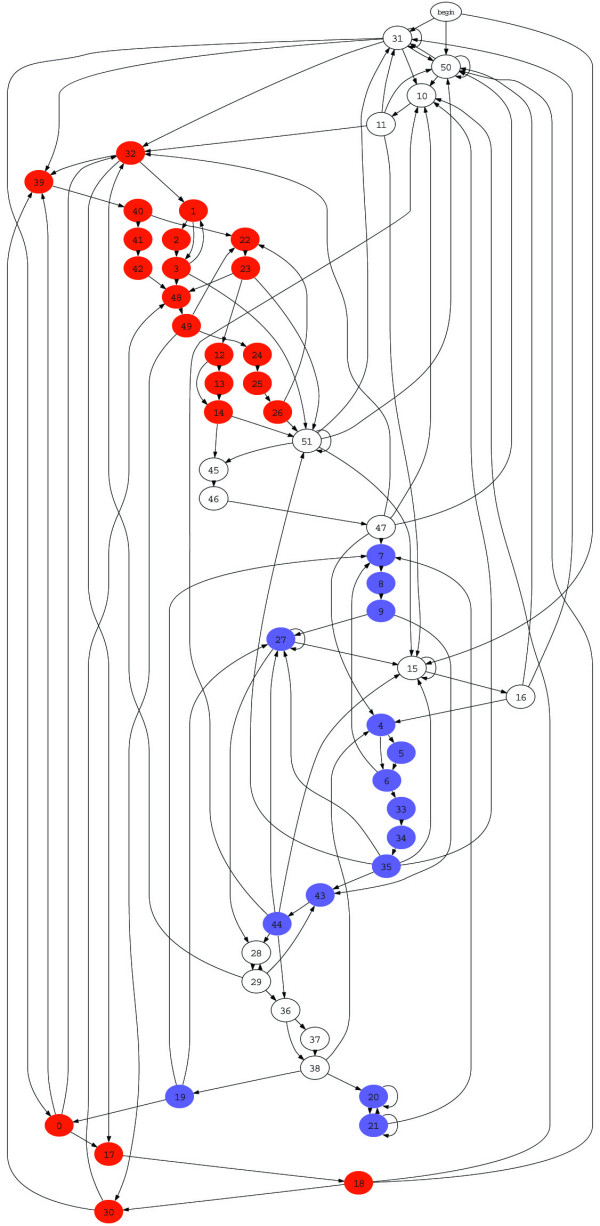
**The full HMM structure**. The full structure of the best HMM topology. Transitions over 0.1 are only shown. States for helix (H), *β*-strand (E) coil (x) are colored with red, blue and white, respectively.

Figure 6 illustrates the structure of the best result of Block-HMMs. The simulation used 30 blocks, but the result shows only 26 blocks: the remaining 4 are zero blocks. Figure 7 shows the full HMM structure. Assigned with each state is one of the label of 3 states of secondary structure *l *∈ {*H*, *E*, *x*}. It is composed of 22 states for helix (*H*), 15 for *β*-strand (*E*), and 15 for coil (*x*) region. Each state emits a set of symbols of 20 amino acids according to the given probability. The full HMM structure is trained using 1662 sequences (see Methods).

The evolved model contains the information of the protein secondary sequences in its structure and parameters. Firstly, we checked the distribution of emission probabilities to see how well the evolved model learned biological information. Table 1 summarizes the characteristics of 51 states, presenting the probabilities of emitting hydrophobic, hydrophilic amino acids, *Gly *and *Pro*. In this table, the linear blocks for *β*-strand (*i.e. state43-state44*, *state7-state8-state9*, *state34-state35*) shows the periodic hydrophilic and hydrophobic characteristics.

**Table 1:** Information of all the trained states

state		AA	H-phobic	H-philic	G	P
state0	H	1.4%	77.8%	21.7%	0.5%	0.0%
state1	H	1.3%	65.3%	24.6%	8.6%	1.5%
state2	H	0.9%	80.1%	14.2%	4.8%	0.9%
state3	H	1.3%	64.6%	27.4%	7.4%	0.6%
state4	E	2.1%	36.6%	53.2%	4.8%	5.5%
state5	E	0.5%	70.3%	28.8%	0.2%	0.7%
state6	E	2.1%	90.4%	9.2%	0.3%	0.1%
state7	E	1.7%	92.7%	1.5%	5.5%	0.3%
state8	E	1.6%	48.5%	47.8%	3.3%	0.5%
state9	E	1.7%	84.8%	10.8%	3.8%	0.6%
state10	x	2.8%	82.2%	17.8%	0.0%	0.0%
state11	x	2.8%	8.7%	50.8%	12.8%	27.7%
state12	H	0.9%	16.3%	79.4%	1.8%	2.5%
state13	H	0.7%	53.8%	44.9%	1.2%	0.2%
state14	H	0.9%	86.1%	13.8%	0.0%	0.0%
state15	x	10.5%	26.1%	50.7%	10.1%	13.1%
state16	x	2.9%	24.9%	45.9%	16.3%	13.0%
state17	H	1.5%	27.1%	62.8%	7.7%	2.3%
state18	H	1.5%	35.7%	59.3%	5.0%	0.0%
state19	E	1.0%	28.1%	56.2%	6.2%	9.5%
state20	E	1.5%	66.4%	27.3%	5.1%	1.2%
state21	E	1.1%	11.8%	75.0%	11.0%	2.2%
state22	H	2.2%	97.8%	2.1%	0.1%	0.0%
state23	H	2.2%	43.2%	51.1%	5.5%	0.1%
state24	H	1.2%	92.4%	6.8%	0.7%	0.1%
state25	H	1.2%	38.9%	60.1%	0.8%	0.2%
state26	H	1.2%	19.3%	79.0%	1.7%	0.0%
state27	E	2.4%	62.0%	33.0%	4.9%	0.1%
state28	x	2.0%	24.7%	54.8%	12.6%	7.9%
state29	x	2.0%	29.6%	45.0%	17.1%	8.4%
state30	H	1.3%	75.4%	20.8%	3.7%	0.0%
state31	x	4.6%	22.5%	63.0%	6.1%	8.5%
state32	H	1.8%	20.2%	45.7%	10.5%	23.5%
state33	E	1.0%	63.2%	33.7%	2.3%	0.8%
state34	E	1.0%	95.4%	2.9%	1.7%	0.0%
state35	E	1.0%	18.0%	65.5%	11.0%	5.5%
state36	x	1.6%	23.6%	65.9%	7.4%	3.1%
state37	x	1.4%	3.5%	40.3%	53.7%	2.5%
state38	x	1.6%	30.0%	57.4%	11.2%	1.4%
state39	H	1.7%	15.7%	71.9%	2.8%	9.6%
state40	H	1.7%	27.8%	67.1%	2.7%	2.3%
state41	H	1.5%	76.5%	21.0%	2.6%	0.0%
state42	H	1.4%	58.7%	40.8%	0.2%	0.2%
state43	E	2.0%	60.4%	34.5%	5.1%	0.0%
state44	E	2.0%	30.5%	57.0%	5.6%	6.9%
state45	x	0.6%	0.6%	35.1%	64.3%	0.0%
state46	x	0.6%	77.6%	19.4%	0.0%	2.9%
state47	x	0.6%	14.6%	71.2%	2.1%	12.2%
state48	H	3.6%	21.9%	74.3%	3.0%	0.7%
state49	H	3.5%	62.1%	34.9%	3.0%	0.1%
state50	x	4.7%	51.4%	32.0%	12.9%	3.7%
state51	x	3.2%	27.6%	57.0%	15.4%	0.0%

At *state11 *and *state32 *we found a strong probability of *Pro*. Among 13637 visits on *state11 *we found *Pro *3765 times (= 27.6%) in the generated sequences, which closely matches with the emission probability of 27.7%. *State11 *usually modelled 'xxx' (2783 times, 73.9%), 'xxH' (685 times, 18.2%), 'xxE' (286 times, 7.6%), and at the end of the sequence ('xx', 11 times). This indicates that *state11 *is used to link a coil with other compositions. In the case of *state 32*, *Pro *was usually used to model 'xHH' (1828 cases out of 2084, 87.7%) or 'HHH' (205 cases out of 2084, 9.8%).

*Gly *was found strong on *state37 *and *state45*. We found *Gly *on *state37 *is only between two coil conformations (3570 times). *Gly *on *state45 *worked in the apposite way to *Pro *on *state11*, producing 'Hxx' (710 times out of 2018, 35.2%), 'xxx' (1300 times, 64.4%), 'Exx' (2 times 0.1%), and at the end of the sequences.

**Figure 8:**
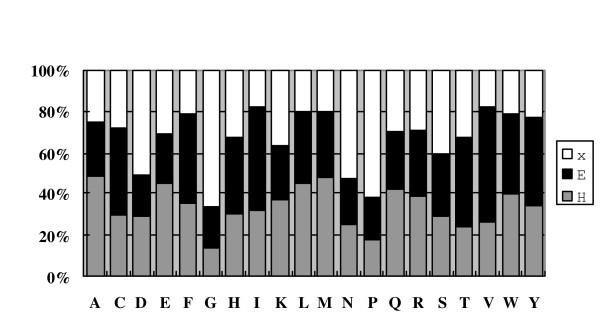
**The averaged emission probabilities of all the states**. The averaged emission probabilities of all the states. Emission probabilities from the states that share the same secondary structural label are averaged.

We examined overall distribution of the emission probabilities in the evolved HMM. We averaged the emission probabilities of all the states assigned to the same secondary label. Figure 8 shows the average distribution of emission probabilities for helix, *β*-strand and coil states. For helices *Ala *and *Leu *are stronger than other amino acids. *Gly *and *Pro *are shown prominently in coils and *Val *is strong in *β*-strands.

#### The HMM's grammar

**Figure 9:**
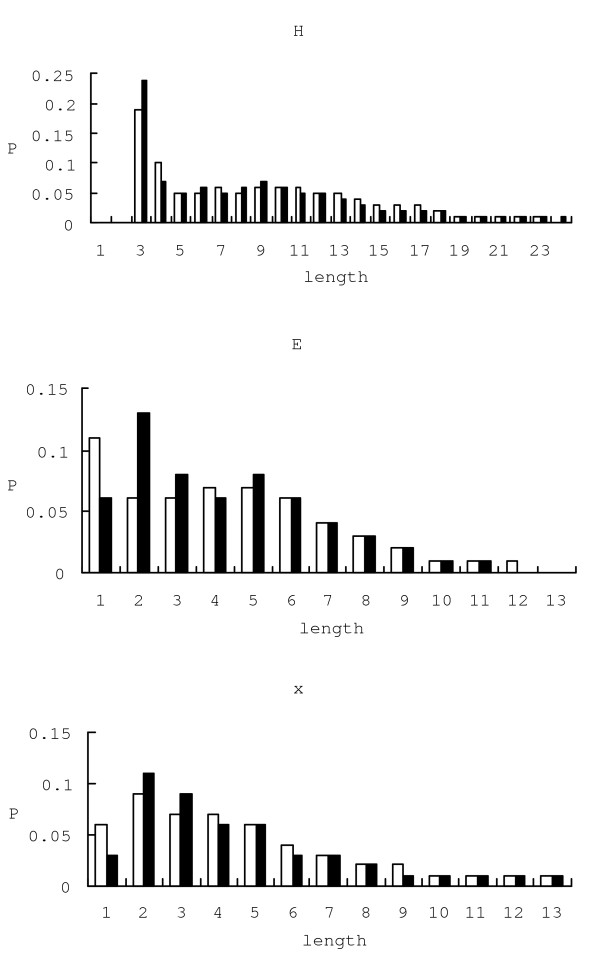
**Histograms of secondary structure element length**. Histograms of the lengths of the secondary structure elements in the training set (white bars) and the generated set (black bars). It shows the probabilities of secondary structure element lengths in the generated sequence.

We evaluated how well the evolved HMM models general features of protein structure. We generated 1662 (the number of training sequences) random sequences from the evolved HMM. We set the length of the generated sequences to be the average length of the training sequences. The third column of Table 2 shows how much each state is used to generate the random sequence. In the generated sequence the overall secondary structure contents are 35.5% of helices, 23.5% of *β*-strands, and 44.5% of coils. This shows that the evolved HMM closely remodels the training sequences composed of 35.3% of helices, 22.8% of *β*-strands, and 41.9% of coils. Figure 9 shows the length distributions of helices, *β*-strands, and coils in the training dataset and the generated set. The distributions closely match each other for three of the cases. The length distribution confirms that a block or a group of blocks model the grammar of protein secondary structure quite closely. We checked how the evolved HMM expresses the grammar of protein sequence in its structure. From the generated sequences we counted the transitions from one block to the other blocks. Table 2 shows summarizes the number of times each block transition is used in the generated sequences and the probability of the transition to be made on each state. We showed only dominant grammars that have been visited more than 2000 times. This result shows how the blocks are used to model the sequences. *State0 *is in a helix block and usually used with other helix blocks (*state17 *and *state39 *are in helix block). *State14 *has a strong transition from helix to coil and *state23 *has transitions to a helix block and a coil block. As a whole the transitions that link blocks with the same label are dominant. This seems to be because the HMM needs to model long secondary elements with very short blocks.

**Table 2:** The block transition

block transition	percentage used on each state	number of times used in the generated sequence
state0 (H) → state17 (H)	36%	2468
state0 (H) → state39 (H)	42%	2867
state3 (H) → state1 (H)	56%	3477
state6 (E) → state33 (E)	24%	2461
state9 (E) → state27 (E)	30%	2377
state9 (E) → state43 (E)	33%	2635
state11 (x) → state15 (x)	30%	4143
state11 (x) → state31 (x)	15%	2093
state11 (x) → state50 (x)	16%	2165
state14 (H) → state51 (x)	60%	2733
state16 (x) → state4 (E)	24%	3310
state16 (x) → state31 (x)	19%	2662
state16 (x) → state50 (x)	35%	4809
state18 (H) → state50 (x)	33%	2359
state19 (E) → state7 (E)	42%	2086
state21 (E) → state7 (E)	55%	2702
state23 (H) → state12 (H)	31%	3347
state23 (H) → state48 (H)	41%	4436
state23 (H) → state51 (x)	21%	2272
state26 (H) → state51 (x)	57%	3394
state27 (E) → state15 (x)	26%	3005
state27 (E) → state28 (x)	34%	3914
state29 (x) → state28 (x)	22%	2193
state29 (x) → state36 (x)	31%	3017
state30 (H) → state48 (H)	75%	4577
state31 (x) → state0 (H)	21%	4564
state31 (x) → state10 (x)	12%	2588
state31 (x) → state31 (x)	16%	3555
state31 (x) → state32 (H)	21%	4551
state31 (x) → state39 (H)	11%	2408
state31 (x) → state50 (x)	13%	2822
state32 (H) → state17 (H)	45%	4062
state38 (x) → state4 (E)	42%	3304
state40 (H) → state41 (H)	66%	5482
state42 (H) → state48 (H)	87%	6105
state44 (E) → state27 (E)	30%	2996
state49 (H) → state22 (H)	39%	6674
state49 (H) → state24 (H)	26%	4508
state49 (H) → state30 (H)	18%	3162
state50 (x) → state10 (x)	13%	2807
state50 (x) → state31 (x)	34%	7362
state50 (x) → state50 (x)	17%	3772
state51 (x) → state15 (x)	14%	2056
state51 (x) → state31 (x)	25%	3597
state51 (x) → state50 (x)	20%	2986
state51 (x) → state51 (x)	20%	2892

We checked if the model has grammar for short secondary elements. We found 57 'H-x-H' linked helices. For this sequence grammar we found an HMM grammar of '*state18*-*state50*-*state32*' 44 times (77.2%). We also checked how each coil-state contribute to this grammar. For the coil region *State50*, *state51 *and *state31 *are used 44 time, 6 time and 7 times, respectively. In the case of 666 'H-x-E' in the generated sequences, the dominant grammars is '*state18*-*state50*-*state43*' (27.6%). For the coil region *State51 *and *State50 *were used 90 times (13.5%) and 576 times (86.5%), respectively. Interestingly, *state31 *was not used for this grammar. For the grammar 'E-x-H', however, *state51 *was never used on the other hand. About 97.2% of the HMM grammar uses *state31 *(923 times out of 950) and 2.8% (27 times) was used by *state50*. This indicates that *state50*, *state51 *and *state31 *are used in a different way when they compose a sequence grammar. For the grammar 'H-xx-H', the dominant HMM grammar used for coil region was '*state51*-*state31*' (1175 times out of 2146 (54.8%)), '*state50*-*state31*' (19.5%) and '*state10*-*state11*' (22.2%). We checked how the HMM is organized to model hairpin structures. For a grammar 'E-x-E', *state50 *was found dominant (81.1% = 310 out of 382). *State51 *and *state31 *covered 3.7% and 15.2%, respectively. For the structure 'E-xx-E', '*state28*-*state29*' are mostly used (58.2% = 1830 out of 3142), followed by '*state15*-*state16*' (16.9%) and '*state36*-*state38*' (11.0%). The single state blocks (*state50*, *state51 *and *state31*) are instead rarely used. In the case of the structure 'E-xxx-E', '*state36*-*state37*-*state38*' covered 68.2% (1937 out of 2842) and *state15*-*state15*-*state16 *occupied 14.0%. Each of other compositions is less than 5%. We generated no sequence for the grammar 'x-H-x', which disobeys the grammar of protein secondary structure.

### Prediction results with posterior decoding

The HMM predictor evolved using Block-HMM method calculates the probability of being in each secondary state. The posterior label probability (PLP) calculates probability of a label of each amino acid. The PLP of a label at position *t *is the sum of posterior probability of all states that emit the same label. The PLP for label *l *∈ {*H*, *E*, *C*} at position *t *is

P(yt=l|x,Θ)=∑i∈Qp(yt=l,qt=i|x,Θ).
 MathType@MTEF@5@5@+=feaafiart1ev1aaatCvAUfKttLearuWrP9MDH5MBPbIqV92AaeXatLxBI9gBaebbnrfifHhDYfgasaacH8akY=wiFfYdH8Gipec8Eeeu0xXdbba9frFj0=OqFfea0dXdd9vqai=hGuQ8kuc9pgc9s8qqaq=dirpe0xb9q8qiLsFr0=vr0=vr0dc8meaabaqaciaacaGaaeqabaqabeGadaaakeaacqWGqbaucqGGOaakcqWG5bqEdaWgaaWcbaGaemiDaqhabeaakiabg2da9iabdYgaSjabcYha8Hqabiab=Hha4jabcYcaSiabfI5arjabcMcaPiabg2da9maaqafabaGaemiCaaNaeiikaGIaemyEaK3aaSbaaSqaaiabdsha0bqabaGccqGH9aqpcqWGSbaBcqGGSaalcqWGXbqCdaWgaaWcbaGaemiDaqhabeaakiabg2da9iabdMgaPjabcYha8jab=Hha4jabcYcaSiabfI5arjabcMcaPaWcbaGaemyAaKMaeyicI48enfgDOvwBHrxAJfwnHbqeg0uy0HwzTfgDPnwy1aaceaGae4heXhfabeqdcqGHris5aOGaeiOla4caaa@6132@

where **x **is an amino acid sequence and **y **is a accompanying sequence labels of protein secondary structure conformation. Θ is the evolved HMM, and Q
 MathType@MTEF@5@5@+=feaafiart1ev1aaatCvAUfKttLearuWrP9MDH5MBPbIqV92AaeXatLxBI9gBaebbnrfifHhDYfgasaacH8akY=wiFfYdH8Gipec8Eeeu0xXdbba9frFj0=OqFfea0dXdd9vqai=hGuQ8kuc9pgc9s8qqaq=dirpe0xb9q8qiLsFr0=vr0=vr0dc8meaabaqaciaacaGaaeqabaqabeGadaaakeaat0uy0HwzTfgDPnwy1egaryqtHrhAL1wy0L2yHvdaiqaacqWFqeFuaaa@3840@ is the set of all the states in the HMM.

We assign each state to one of the classes in the secondary structure. That is, we take the probability of a label given a state to be 1 if the state is assigned to that class and 0 otherwise. Thus the sum in equation (1) only gets contributions from states that have been assigned to class *l*.

**Figure 10:**
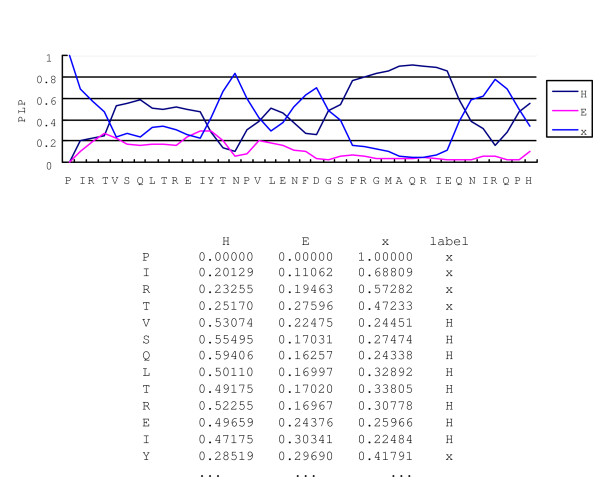
**The decoding result with posterior decoding**. The decoding result with posterior decoding. The PLP calculates probability of a label of each amino acid. The dominant label is assigned as a final prediction

Figure 10 shows the PLP value along part of a protein sequence *1ciy*. The probability of each label is calculated and drawn in the graph. The dominant label is assigned to each amino acid as a prediction result.

### Prediction under single-sequence condition

#### Cross-validation results

We conducted 5 cross-validation tests with very stringent dataset conditions (see Methods). By running Block-HMM we evolved HMM structures separately from each of the cross-validation test. Under the single-sequence condition, we achieved a overall prediction rate (*Q*_Ì_) of 68.3% using a single HMM predictor (Table 3).

**Table 3:** Prediction under the single-sequence condition

Test	*Q*_Ì_	*Q*_*H*_	*Q*_*E*_	*Q*_*C*_	*SOV*	*SOV*_*H*_	*SOV*_*E*_	*SOV*_*C*_
5-fold cross-validation	68.3	65.9	56.4	74.8	63.9	63.8	59.8	65.8
Non-common (Block-HMM)	68.6	67.6	58.0	74.1	64.1	64.9	61.2	65.4
Non-common (PSIPRED)	67.3	65.8	58.9	70.5	63.6	64.2	60.7	63.1
Common (Block-HMM)	69.0	66.1	56.6	76.3	63.6	63.4	59.8	66.7
Common (PSIPRED)	67.6	63.4	56.0	73.8	63.1	62.8	58.2	63.8

#### Prediction comparison

We compared performance of the best HMM topology trained on all the 1662 training sequences with other predictors under the single-sequence condition. As a test set we used the data set published on October 2002 on the EVA server [31]. From this we prepared two sets. Firstly, from 1828 sequences we deleted the common sequences with our training set and finally retrieved 1584 sequences (non-common set). Secondly, we only used the sequences which are common in our training set and PSIPRED training set and found 153 sequences (common set). Table 3 shows the comparison with PSIPRED for the two tests. These tests at least show that Block-HMM has good performance as a secondary structure predictor.

### Prediction under multi-sequence condition

We designed a whole secondary structure predictor using multiple sequences information. Structure-to-Structure layer is added to get more prediction. See Methods for more detail.

#### Cross-validation & comparison

Table 4 shows the result of 5 cross-validation tests. By using multiple sequence alignment the *Q*_Ì _value increased about 6.8% (68.3% under single-sequence condition).

**Table 4:** Prediction under the multiple sequences condition

Test	*Q*_Ì_	*Q*_*H*_	*Q*_*E*_	*Q*_*C*_	*SOV*	*SOV*_*H*_	*SOV*_*E*_	*SOV*_*C*_
5-fold cross-validation	75.1	67.8	70.8	77.5	71.7	68.4	73.4	69.6
Non-Common (PSIPRED)	78.9	76.7	74.5	77.3	75.6	76.3	75.6	71.3
Non-Common (YASPIN)	73.4	68.8	83.0	68.9	71.1	70.1	76.5	65.8
Non-Common (BLOCK-HMM)	74.5	70.3	69.6	76.2	70.6	69.5	72.7	68.2
Common (PSIPRED)	79.5	74.6	71.7	79.6	75.8	74.4	73.2	72.6
Common (YASPIN)	74.6	68.2	80.1	71.0	71.3	68.4	74.7	67.2
Common (BLOCK-HMM)	75.0	67.4	67.2	78.7	70.5	67.7	68.6	69.7

In an attempt to benchmark our method with existing predictors we compare our prediction results with those of YASPIN [10] and PSIPRED. We asked Dr. Kuang Lin to train YASPIN with the same data set we used. The same dataset is used in running PSIPRED which has been already trained and publicly available. We used 2 data sets used in the test under the single-sequence condition. Table 4 shows the benchmarking result. When we used non-common dataset the *Q*_Ì _rate of our method is about 1% better than that of YASPIN, though the *SOV *of YASPIN is about 0.5% higher. The *Q*_*E *_of YASPIN is impressive, showing better performance than PSIPRED. Obviously, PSIPRED showed best performance. Next, we used the common set. Again, PSIPRED showed best performance, and the performance of Block-HMM is about 1% better than YASPIN. This result is interesting, considering that the performance of Block-HMM is better using same dataset under the single-sequence condition.

## Discussion

The predictor using HMMs inherited all the advantages of HMMs. Artificial protein sequence with secondary structure can be generated. The generated sequences show matched characteristics with the training dataset in the contents and the length distributions of the secondary conformation. Also, it is easy to see probabilistic reasoning of the prediction result. The analysis on the evolved model and the generated sequences shows that the evolving method successfully interprets the grammar of the protein sequences and converts it into the grammar of HMMs. It is more noteworthy considering that the grammar and biological information is constructed automatically without human intervention.

Recently, an HMM based protein secondary predictor was hand designed and showed good performance in predicting *beta*-strands under single-sequence condition [32]. Also, structure learning method using *Bayesian information criterion *has been introduced [33]. It increases the number of states while checking the optimal balance between fitting to the data and the HMM size. Our method has more operations to change HMM structure and we penalised the number of HMM structure by evaluating the trained model with the separated set. As shown in the test under the single-sequence condition, the overall prediction performance of an evolved HMM is quite excellent. We do not claim that our HMM is better under the single-sequence condition. The test set we used may be biased to HMMs. However, the result at least shows that the evolving method is a good way to design an HMM for this problem and further applications. In the case of testing under the multiple sequences condition, the performance of PSIPRED is obviously better than Block-HMM. The way of incorporating multiple sequences information as well as the structure-to-structure layer of PSIPRED works far better than our approach. Incorporating multiple sequences information remains further area of study. However, our result still comparable to YASPIN's result.

Our method does not require a sliding window as most other secondary structure prediction methods do. The size of the window is chosen in order to obtain good performance (for example, PSIPRED has a window size of 15 [7]). The evolving HMM method uses the whole sequence as input, which avoids the use of a fixed sequence window that might affect performance in specific cases.

At present the Block-HMM method is relatively slow because it has to train and calculate fitness for all the HMM members in the population. Fortunately, the method is suitable for parallel computation. To evolve an HMM using GAs with 30 members in a population, we used 31 2.4 GHz P4 processors each with 512 Mb RAM run in parallel. Each processor trains one HMM. Ideally, the CPU time consumed in each processor is the time to train and evaluate an HMM multiplied by the number of iterations. It took about 7 hours to produce an HMM with 40 states. Prediction using three trained HMMs without evolutionary information takes about 30 seconds.

## Conclusion

Optimizing HMM structures using an evolutionary algorithm has several benefits. First of all, the structure of an HMM is automatically evolved without prior knowledge. The success is remarkable given that other methods for secondary structure prediction require considerable calibration. Compared to the hand-designed HMMSTR [19], the evolutionary method produced good results with a smaller number of states. In the case of neural networks, the selection of the number of units needs careful attention. Here again, the evolving HMM method is an attractive alternative.

Compared to other HMM structure evolving methods, our approach shows excellences. Thomsen's results for the secondary structure prediction (49%) indirectly tells that our method is very effective for the secondary structure prediction problem.

The P.S.HMM (Protein Secondary structure predictor using HMMs)server is online, providing secondary structure prediction and probability of each secondary structure conformation. Protein dataset used in the test is found at http://binf.ku.dk/~won/proseq.tar.gz.

## Methods

### Data set

The SABMark Twilight Zone data set (version 1.63) [34] provides a set of representative structures. This data set consists of 2230 high quality structures partitioned into 236 folds. Although many proteins in the data set share a common fold, no pair of protein sequences can be aligned with a BLAST E-value below 1 or a sequence identity above 25%. For the proteins with a common fold in the data set, it is not possible to identify a traceable evolutionary common origin.

Structures that caused problems with the DSSP program (see below) or that had chain breaks were removed, which resulted in a final data set of 1662 structures belonging to 234 fold groups. Two fold groups are removed by this process because no structures remained in these groups. With these 234 groups we performed a five fold cross-validation test. In order to create a stringent test set we made sure that proteins with a common fold do not appear in both the training and test sets.

The secondary structure was calculated using the program DSSP [35]. DSSP assigns secondary structure to eight different classes: *α*-helix (H), isolated *β*-bridge (B), *β*-strand (E), 3_10_-helix (G), Π-helix (I), turn (T), bend (S) and other. In this study, we used three classes: helix (consisting of DSSP classes H and G), strand (classes B and E) and coil (all other classes). The DSSP results were retrieved using the DSSP front end in the Biopython toolkit [36].

### Training with Block-HMM

We have used a hybrid GA with traditional GA operators to explore the space of HMM topologies in combination with Baum-Welch optimization of the transition and emission probabilities.

To obtain suitable HMM architectures we tested various numbers of blocks between 26 and 35. Labels are allocated randomly to each of the blocks. The size of the block, that is the number of states in a block, is randomly assigned between 1 and 4. Table 5 shows the parameters used in the simulation.

**Table 5:** Block-HMM parameters used in the experiment

Parameter	value
Population size	30
Iteration	400
Number of blocks in an HMM	26–35
The initial length of a block	1–4
Number of crossovers per iteration	2
Number of mutations per iteration	2
Number of type-mutations per iteration	2

To find an HMM that does not overfit the training data, we divide our training set into a set used for the Baum-Welch training (5/7 of the data) and a set for fitness evaluation (2/7 of the data). The fitness value is calculated from the fitness evaluation set only. Given an HMM (with parameters Θ), we take the reciprocal of the negative log-likelihood as the fitness value:

Eμ=1−∑ilog⁡(P(xi|Θμ))/li
 MathType@MTEF@5@5@+=feaafiart1ev1aaatCvAUfKttLearuWrP9MDH5MBPbIqV92AaeXatLxBI9gBaebbnrfifHhDYfgasaacH8akY=wiFfYdH8Gipec8Eeeu0xXdbba9frFj0=OqFfea0dXdd9vqai=hGuQ8kuc9pgc9s8qqaq=dirpe0xb9q8qiLsFr0=vr0=vr0dc8meaabaqaciaacaGaaeqabaqabeGadaaakeaacqWGfbqrdaWgaaWcbaacciGae8hVd0gabeaakiabg2da9maalaaabaGaeGymaedabaGaeyOeI0YaaabeaeaacyGGSbaBcqGGVbWBcqGGNbWzcqGGOaakcqWGqbaucqGGOaakcqWG4baEdaWgaaWcbaGaemyAaKgabeaakiabcYha8jabfI5arnaaBaaaleaacqWF8oqBaeqaaOGaeiykaKIaeiykaKIaei4la8IaemiBaW2aaSbaaSqaaiabdMgaPbqabaaabaGaemyAaKgabeqdcqGHris5aaaaaaa@4A39@

where *l*_*i *_is the length of a sequence *x*_*i *_and *μ *labels the different HMMs (with parameters Θ_*μ*_) of the population. A member of the population is selected with a Boltzmann probability

Fμ=mμ∑ν=1Nmν,mμ=esEμ/σ
 MathType@MTEF@5@5@+=feaafiart1ev1aaatCvAUfKttLearuWrP9MDH5MBPbIqV92AaeXatLxBI9gBaebbnrfifHhDYfgasaacH8akY=wiFfYdH8Gipec8Eeeu0xXdbba9frFj0=OqFfea0dXdd9vqai=hGuQ8kuc9pgc9s8qqaq=dirpe0xb9q8qiLsFr0=vr0=vr0dc8meaabaqaciaacaGaaeqabaqabeGadaaakeaafaqabeqacaaabaGaemOray0aaSbaaSqaaGGaciab=X7aTbqabaGccqGH9aqpdaWcaaqaaiabd2gaTnaaBaaaleaacqWF8oqBaeqaaaGcbaWaaabmaeaacqWGTbqBdaWgaaWcbaGae8xVd4gabeaaaeaacqWF9oGBcqGH9aqpcqaIXaqmaeaacqWGobGta0GaeyyeIuoaaaGccqGGSaalaeaacqWGTbqBdaWgaaWcbaGae8hVd0gabeaakiabg2da9iabbwgaLnaaCaaaleqabaGaem4CamNaemyrau0aaSbaaWqaaiab=X7aTbqabaWccqGGVaWlcqWFdpWCaaaaaaaa@4BEF@

where *σ *is the standard deviation of the fitness in the population and *s *is a constant that controls the strength of the selection. In the work reported here, we used a value of *s *equal to 0.3.

The best member of a population is always selected, and a subset of other members are selected by using stochastic universal sampling [37]. Some of the members are mutated or subjected to crossover. Then, all the members of the generation undergo Baum-Welch optimization using the training data set.

We saved the best HMM at each of the 400 generations, *i.e. *during the whole run of the GA. At the end of the run, the best HMM is selected and trained again with the Baum-Welch algorithm, this time using all the sequences used for training and evaluation. This is done because the last HMM is not always the best HMM generated during the whole GA run. Finally, the HMM is trained further using the discriminative training method [38]. The Baum-Welch algorithm maximizes the likelihood of the training sequences (in our case containing amino acid and secondary structure labels). However, we are more interested in maximizing the probability of obtaining correct secondary structure labels for the amino acid sequences (rather than maximizing the probability of the full sequences themselves). Discriminative training is used to increase the probability of obtaining correct labels given the sequences and a specific HMM structure.

### Incorporating evolutionary information

Secondary structure prediction rates can be boosted by using evolutionary information. In most systems, the position specific scoring matrix (PSSM) is used as an input of the predictor. Instead of using PSSM, we ran our predictor on a set of homologous sequences and then combined the results. To obtain the homologous sequences we ran PSI-BLAST [14] against the UniProt 90 protein sequence database [39] downloaded on Feb. 17th 2005. We used 3 iterations of PSI-BLAST and an E-value threshold of 0.001. The posterior label probabilities (PLPs) were calculated by decoding each of the homologous sequences against the trained HMM. After aligning the decoding results, we calculated the weight of each sequence according to the position-based sequence weight [40].

### The second (structure-to-structure) layer

**Figure 11:**
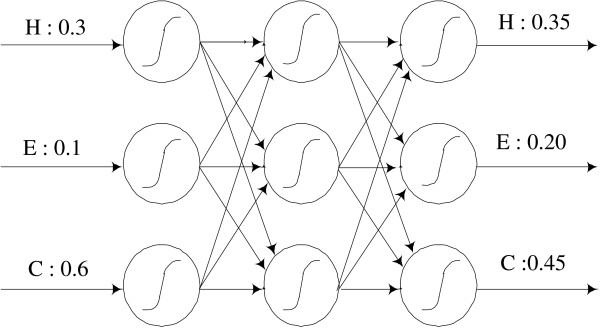
**The structure-to-structure layer**. The structure-to-structure layer is composed of simple 3-layer neural networks.

To improve the performance even further we used a 3-layer perceptron consisting of 3 input nodes, 3 hidden nodes and 3 output nodes. This network is shown in figure 11. The profile averaged PLPs of the HMM are used directly as input to the neural network. This network is quite simple compared to other structure-to-structure layers published in the literature. To train the neural networks the gradient descent method with a momentum term was used [41].

**Figure 12:**
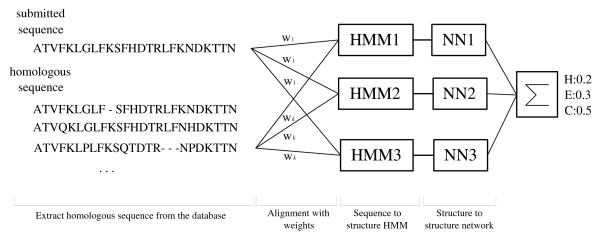
**Overview of protein secondary structure predictor**. Schematic overview of predicting secondary structure with three HMMs evolved with Block-HMM.

To increase the prediction rate further we used an ensemble of three independently trained HMM predictors. The three HMM structures are different because they were found by different runs of Block-HMM. This approach improves the prediction rate more than combining HMMs that have the same structure but different parameters. The outputs of the structure-to-structure layer are summed up and the dominant label is used as our final prediction of the secondary structure. The final predictor is shown in figure 12.

## Competing interests

The author(s) declares that there are no competing interests.

## Authors' contributions

KJW implemented the algorithms, performed all tests, and made all images. TH constructed the protein data sets, provided advice on protein structure and contributed to the analysis of the results. APB and AK conceived of the algorithm and participated in its design and coordination. KJW, TH, APB and AK wrote the manuscript. All authors read and approved the final manuscript.
